# LYN expression predicts the response to dasatinib in a subpopulation of lung adenocarcinoma patients

**DOI:** 10.18632/oncotarget.12657

**Published:** 2016-10-14

**Authors:** Yu Jin Kim, Sungyoul Hong, Minjung Sung, Min Jeong Park, Kyungsoo Jung, Ka-Won Noh, Doo-Yi Oh, Mi-Sook Lee, Ensel Oh, Young Kee Shin, Yoon-La Choi

**Affiliations:** ^1^ Laboratory of Cancer Genomics and Molecular Pathology, Samsung Medical Center, Sungkyunkwan University School of Medicine, Seoul, Korea; ^2^ Laboratory of Molecular Pathology and Cancer Genomics, Department of Pharmacy, College of Pharmacy, Seoul National University, Seoul, Korea; ^3^ Department of Health Sciences and Technology, SAIHST, Sungkyunkwan University, Seoul, Korea; ^4^ The Center for Anti-cancer Companion Diagnostics, Bio-MAX/N-Bio, Seoul National University, Seoul, Korea; ^5^ Department of Pathology and Translational Genomics, Samsung Medical Center, Sungkyunkwan University School of Medicine, Seoul, Korea

**Keywords:** LYN, dasatinib, lung adenocarcinoma subgroup, SRC, YES

## Abstract

Therapies targeting SRC family kinases (SFKs) have shown efficacy in treating non-small cell lung cancer (NSCLC). However, recent clinical trials have found that the SFK inhibitor dasatinib is ineffective in some patient cohorts. Regardless, dasatinib treatment may benefit some NSCLC patient subgroups. Here, we investigated whether expression of LYN, a member of the SFK family, is associated with patient survival, the efficacy of dasatinib, and/or NSCLC cell viability. LYN expression was associated with poor overall survival in a multivariate analysis, and this association was strongest in non-smoker female patients with adenocarcinoma (ADC). In lung ADC cells, LYN expression enhanced cell proliferation, migration, and invasion. Dasatinib inhibited LYN activity and decreased cell viability in LYN-positive ADC cell lines and xenografts. Additionally, we identified the SFKs SRC and YES as candidate dasatinib targets in LYN-negative ADC cell lines. Our findings suggest that LYN is a useful prognostic marker and a selective target of dasatinib therapy in the lung ADC subpopulation especially in female non-smokers with lung ADC.

## INTRODUCTION

Lung cancer, the leading cause of cancer-related death worldwide, can be classified as either small cell or non-small cell lung carcinoma (NSCLC). NSCLC accounts for approximately 85% of all lung cancers, and can be further histologically classified as squamous cell carcinoma (SCC), adenocarcinoma (ADC), large cell carcinoma, or undifferentiated [1]. NSCLC is often associated with epidermal growth factor receptor (EGFR) overexpression, which occurs in 40%–80% of patients; therefore, EGFR-targeting therapies have been investigated [2]. However, these therapies are effective only in NSCLC patients with activating EGFR mutations; therefore, alternative therapies are required to target NSCLCs that do not respond to current treatment regimens [3].

SFKs are intracellular membrane-associated non-receptor tyrosine kinases and include SRC, LYN, YES, FYN, BLK, YRK, FGR, HCK, and LCK [4]. The overexpression and activation of SFKs promote the development of tumor malignancy and regulate cell proliferation, survival, and angiogenesis during tumor progression [4, 5]. SFKs are frequently over-expressed and over-activated in NSCLC [4, 6–8], and targeting SFKs in NSCLC is a promising clinical strategy. Dasatinib, a potent SFKs inhibitor, is currently being evaluated in several clinical trials for use in NSCLC. However, these studies, which did not consider genetic information, reported that dasatinib was ineffective in their patient cohorts [9–12]. Therefore, it is of interest to determine whether certain NSCLC patient subgroups may benefit from dasatinib treatment, either as a monotherapy or in combination with other therapies, compared to NSCLC patients in general.

LYN, a major member of the SFK family, is expressed in many solid tumors, including colon cancer, prostate cancer, glioblastoma, and breast cancer [13, 14], and plays a critical role in tumor progression. LYN promotes colorectal cancer development via CD24-mediated ERK1/2 activation, and dasatinib is effective in treating metastatic prostate cancers harboring activated LYN [15, 16]. LYN was suggested as a candidate marker of dasatinib sensitivity based on gene expression signatures from a panel of breast cancer cell lines [17]. We also found that LYN is a potential therapeutic target of dasatinib in clinically aggressive basal-like breast cancer [18]. A recent study showed that inhibition of LYN resulted in synthetic lethality after dasatinib treatment in breast-cancer cell lines expressing high levels of MYC [19]. Moreover, LYN helped maintain NSCLC viability by regulating EGFR [20]. Using a drug-immunoaffinitychromatography approach, LYN was identified as one of the 18 tyrosine kinase targets of dasatinib in a lung mucoepidermoid cancer cell line [21]. In this study, we investigated whether LYN is a selective therapeutic target of dasatinib in the treatment of NSCLC. We examined whether LYN expression predicted clinical outcomes in NSCLC patients to assess its potential as a prognostic marker and therapeutic target. We also investigated the impact of LYN expression on NSCLC viability and evaluated the effects of dasatinib-induced LYN inhibition on cell growth in NSCLC cell lines and xenograft models.

## RESULTS

### Patient characteristics

Clinicopathological features of the enrolled NSCLC patients are summarized in Supplementary Table S1 and grouped according to LYN expression status. There were 360 (80.7%) male and 86 (19.3%) female patients, and the median age at diagnosis was 55 years (range, 20–82 years). In total, 246 (55.2%), 102 (22.8%), and 98 (22.0%) patients were diagnosed with stage I, stage II, and stage III disease, respectively. Of the 446 NSCLC tissues included in this study, 254 (57.0%), 151 (33.8%), and 41 (9.2%) were classified as SCC, ADC, and other, respectively. The majority of patients were current or former smokers (327 patients, 73.3%), while 119 patients (26.7%) had never smoked (non-smoker).

### LYN expression is associated with poor survival in the lung ADC subgroup

To investigate the clinical significance of LYN in NSCLC, we examined LYN expression in NSCLC tissues. LYN protein expression was examined in 446 NSCLC tissues by immunohistochemistry (IHC) using paraffin-embedded tissue microarray (TMA) specimens. LYN activation is regulated by the trans/auto-phosphorylation of Y396, which induces an active configuration [13]. We initially attempted to detect phospho-LYN (Y396) using antibodies from two different companies, but these antibodies did not show specificity for the active LYN form. We then attempted to use a Duolink *in situ* proximity ligation assay from Sigma-Aldrich to detect pY396-LYN, but it was also unable to detect a specific signal. Ultimately, we used a total LYN antibody for the IHC assay.

**Figure 1:**
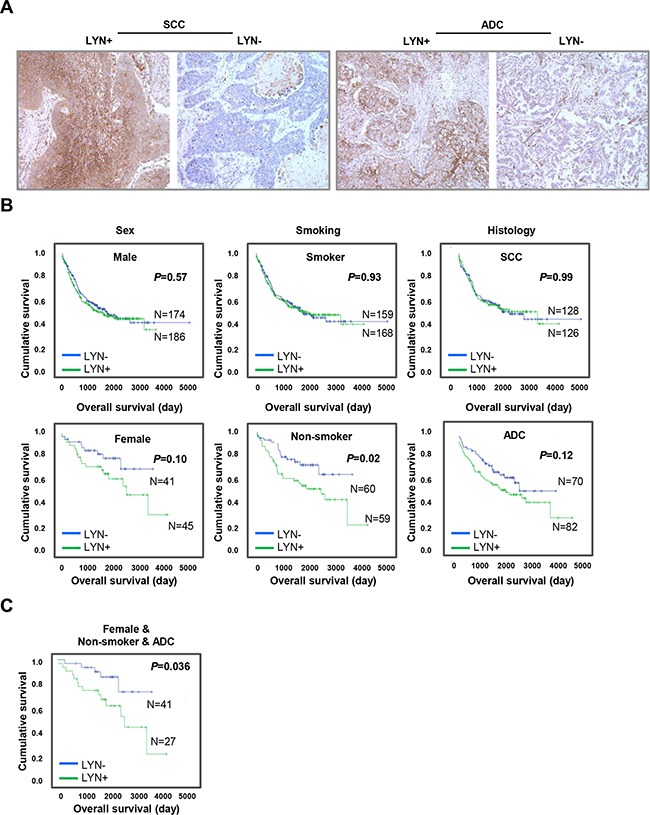
LYN expression is associated with poor clinical outcomes in lung ADC subgroups **A.** LYN expression was examined by immunohistochemical staining of NSCLC patient tissue microarrays. *Left panel*s, positive (left) and negative (right) LYN expression in lung SCC. *Right panels*, positive (left) and negative (right) LYN expression in lung ADC. **B.** The impact of LYN expression on OS in NSCLC patients was analyzed using the Kaplan-Meier method after subgrouping patients by sex, smoking history, and tumor histology. **C.** The impact of LYN expression on OS in the non-smoker females with ADC patient subgroup was determined using the Kaplan-Meier method.

LYN immunostaining indicated positive LYN expression if more than 10% of immunopositive signals were localized near the cell membrane (representative images are shown in Figure 1A). Approximately half of the tissues examined were LYN-positive (227; 50.9%); the rest were LYN-negative (219; 49.1%). There were no significant associations between LYN expression and sex (*P* = 0.293), age (*P* = 0.313), smoking history (*P* = 0.410), tumor histology (*P* = 0.705), lymph node status (*P* = 0.165), or disease stage (*P* = 0.605) (Supplementary Table S1).

Next, we evaluated the prognostic value of LYN expression in predicting overall survival (OS) in NSCLC patients. Although LYN expression was not significantly associated with poor OS in a univariate analysis (HR = 1.186, *P* = 0.228) (Supplementary Table S2), the association was significant in a multivariate analysis (HR = 1.602, *P* = 0.025) (Table 1). We further analyzed the prognostic value of LYN expression by subgrouping patients with respect to sex, smoking history, and tumor histology using Kaplan-Meier analysis. There was no association between LYN expression and OS in males (*P* = 0.57), smokers (*P* = 0.93), or patients with SCC (*P* = 0.99) (Figure 1B, upper panel). By contrast, LYN expression was associated with lower OS in non-smokers (*P* = 0.02); there was a trend towards the same association in females and patients with ADC (*P* = 0.10 and 0.12, respectively) (Figure 1B, lower panel). Additionally, LYN expression was associated with poor OS in the non-smoker females with ADC subgroup (*P* = 0.036) (Figure 1C), but not in the non-smoker males with ADC subgroup (*P* = 0.176, data not shown). Similarly, in Cox multivariate analysis, LYN expression was associated with lower OS in non-smokers (HR = 2.527, *P* = 0.005); there was a trend towards the same association in females (HR = 2.097, *P* = 0.079), but not in patients with ADC (HR = 1.309, *P* = 0.274) (Supplementary Table S3). The association between LYN expression and lower OS was also strongest in the non-smoker females with ADC subgroup (HR = 3.449, *P* = 0.023) in the multivariate analysis (Table 1).

**Table 1:** Multivariate analysis of prognostic factors for overall survival in the entire patient cohort and the female, non-smoker, ADC subgroup

Variable	Category	Total group (n=446)			Female, non-smoker, and ADC (n=68)		
		HR	95% CI	*P*	HR	95% CI	*P*
Sex	Male/Female	1.645	0.943-2.870	0.080	-	-	-
Age group	≥65 yr/<65 yr	1.637	1.231-2.177	<0.01	2.598	0.829-8.136	0.101
Smoking	Smoker/Non-smoker	1.336	0.862-2.070	0.195	-	-	-
Histology	SCC/ADC	1.580	1.140-2.191	0.006	-	-	-
Stage	III/I+II	2.436	1.709-3.473	<0.01	7.755	1.497-10.16	0.015
LYN	Positive/Negative	1.602	1.062-3.419	0.025	3.449	1.182-8.065	0.023

SCC, squamous cell carcinoma; ADC, adenocarcinoma; HR, hazard ratio; CI, confidence interval; *P*, *p*-value

Next, we examined whether specific *EGFR* mutations were associated with non-smoker, female patients with ADC. Deletion of exon 19 and an L858R mutation in exon 21 result in activation of the kinase domain, and a T790M mutation in exon 20 results in resistance to EGFR inhibitors [22, 23]. We PCR amplified and sequenced genomic DNA isolated from 54 formalin-fixed, paraffin-embedded (FFPE) tissues. *EGFR* mutations in exon 19/21 and exon 20 were identified in 41.5% and <1% of the tissues examined, respectively. These mutation rates were consistent with the incidences observed in the general NSCLC patient population, and *EGFR* mutations did not correlate with LYN expression status in non-smoker, female patients with ADC (*P* > 0.05, Supplementary Table S4) [24–26].

### LYN expression drives oncogenic phenotypes in lung ADC cells

**Figure 2:**
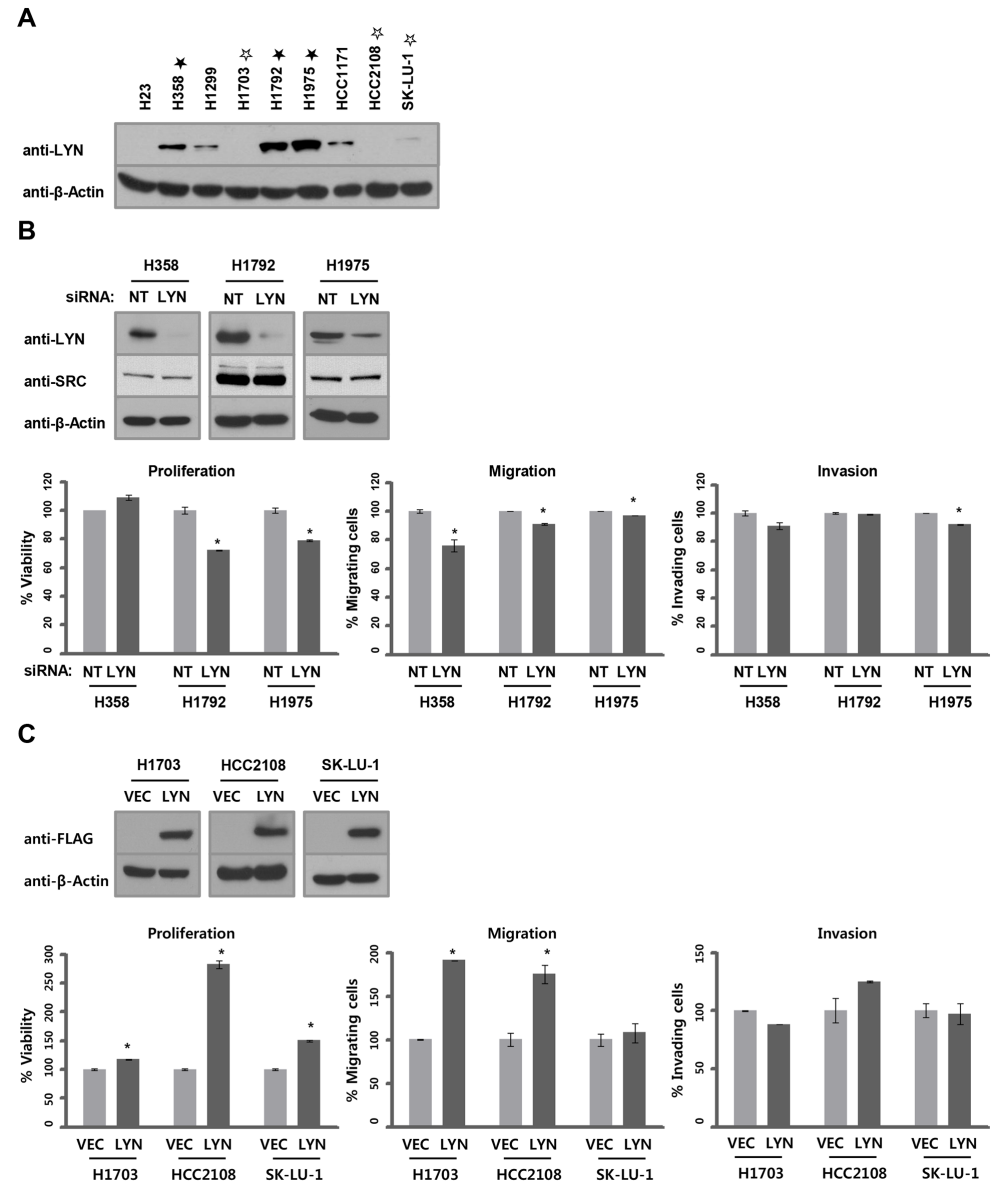
LYN promotes lung ADC progression **A.** LYN protein levels were determined by western blot. **B.** siRNA-mediated LYN knockdown was confirmed by western blot in H358, H1792, and H1975 cells. NT, non-targeting. To verify the specificity of siRNA-mediated LYN knockdown, levels of SRC, a major member of the SFK family, was analyzed by western blot. The effects of LYN knockdown on cell proliferation (left), migration (center), and invasion (right) were evaluated using the WST-1 assay (proliferation) and the Boyden chamber assay (migration and invasion; n = 3; Bars, SE; *, *P* < 0.05; Student's *t*-test). **C.** LYN overexpression was confirmed by western blot in H1703, HCC2108, and SK-LU-1 cells. VEC, empty vector. Phenotypes assayed as above.

Because LYN expression is associated with clinical outcomes in lung adenocarcinoma, and in patients with ADC in general, we examined the functions of LYN in ADC cell lines. We first compared LYN protein levels in ADC cell lines using western blots. Five of nine ADC cell lines had high LYN levels (Figure 2A), which was consistent with our results in the TMA specimens (Supplementary Table S1, 82 of 151 ADC cases, 54.3%). We next examined whether LYN regulates the growth of ADC cells. ADC cell lines expressing high levels of LYN (H358, H1792, and H1975; filled asterisk) were treated with LYN siRNA (or control non-targeting siRNA), and LYN depletion was confirmed using western blots. LYN knockdown reduced cell proliferation (H1792 and H1975; *P* < 0.05), migration (H358, H1792, and H1975; *P* < 0.05), and invasion (H1975; *P* < 0.05) (Figure 2B, Supplementary Figure S2 and Supplementary Table S5). In complementary experiments, ADC cell lines expressing low or undetectable levels of LYN (H1703, HCC2108, and SK-LU-1; empty asterisk) were transfected with LYN expression vectors (or empty vector control), and LYN overexpression was confirmed using western blots. LYN overexpression enhanced cell proliferation (H1703, HCC2108, and SK-LU-1; *P* < 0.05) and cell migration (H1703 and HCC2108; *P* < 0.05), but not invasion (Figure 2C). These results indicate that LYN contributes to tumorigenic phenotypes in lung ADC cells.

We then screened for activating *LYN* mutations, particularly focusing on exons 8, 11, and 12, which correspond to the P-loop and tyrosine kinase domains. Recurrent mutations in these exons (E241D, G264S, Y357C, and K404N), were detected in samples from patients with lung ADC, and were also identified in The Cancer Genome Atlas (https://tcga-data.nci.nih.gov). We PCR amplified and sequenced genomic DNA isolated from 54 FFPE tissues (out of 68 from the non-smoker, female patients with ADC group in Figure 1C). We identified an activating *LYN* mutation (Y357C) in only one LYN-positive sample (*P* > 0.05, Supplementary Table S4), suggesting that *LYN* mutation is not a dominant mechanism of LYN activation in ADCs.

### Lung ADC cells are sensitive to dasatinib

To evaluate whether LYN inhibition affects their growth and viability, ADC cells were treated with dasatinib. First, we attempted to specifically inhibit LYN phosphorylation using bafetinib, a LYN-selective inhibitor, but it was not effective in ADC cells (data not shown). Therefore, we instead treated ADC cells with dasatinib, a general SFK inhibitor. Dasatinib is more effective in NSCLC cells harboring specific, single EGFR inhibitor mutations (deletion of exon 19 or L858R in exon 21), but not in cells with double mutations (deletion of exon 19 and T790M or L858R and T790M) [27–29]. Based on these reports, we tested ADC cells harboring WT *EGFR* and H1975 cells harboring double mutations (T790M and L858R, Supplementary Table S6). The inhibitory concentration (IC_50_) of dasatinib in H23 and SK-LU-1 cells, which had low or undetectable LYN expression (Figure 2A), was 10.68 and 7.86 μM, respectively (Supplementary Table S7 and Supplementary Figure S1A). In contrast, dasatinib had strong inhibitory activity in H358 and H1975 cells, which have high LYN expression, with IC_50_ values of 0.266 and 0.349 μM, respectively (Figure 2A, Supplementary Table S6 and Supplementary Figure S1B).

**Figure 3:**
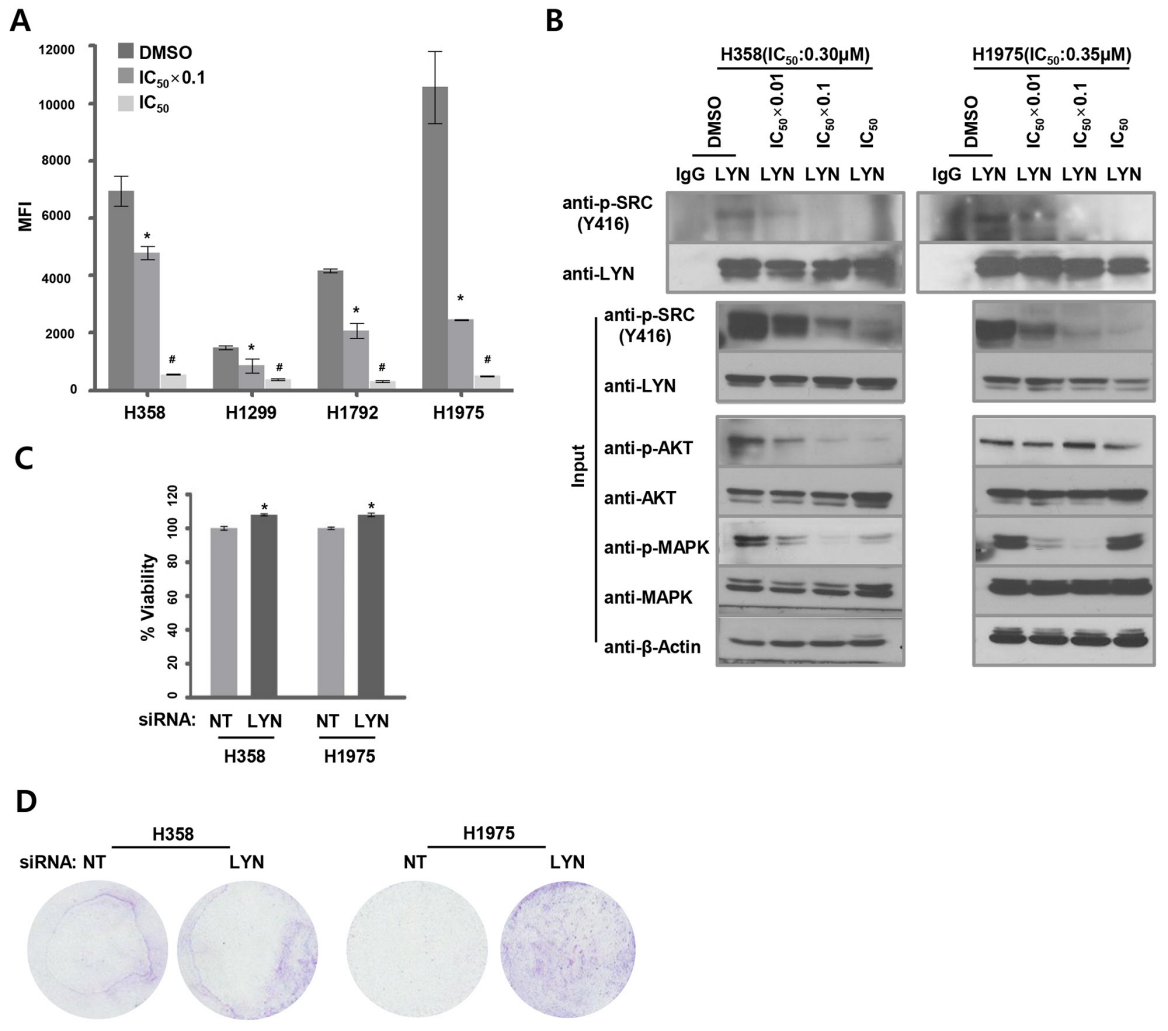
Dasatinib suppresses LYN activity **A.** LYN tyrosine (Y396) phosphorylation levels were quantified using ELISA in dasatinib-treated ADC cells. Cells were treated with dasatinib at 2 different concentrations (DMSO as a control, IC_50_ x 0.1, and IC_50_). Median fluorescence intensity (MFI) values indicate levels of active pY396-LYN (n = 3; *, *P* < 0.05 vs. DMSO; ^#^, *P* < 0.05 vs. IC_50_ x 0.1). **B.** Activated pY396-LYN levels were also measured by immunoprecipitating lysates from H358 and H1975 cells with an LYN antibody and immunoblotting with a cross-reactive pY416-SRC antibody. Cells were treated with dasatinib at 3 concentrations, and activated AKT and MAPK levels were determined using western blots. **C.** Cells were treated with dasatinib (DMSO as a control, IC_50_ × 0.1) 24 hrs after siRNA-mediated LYN knockdown. The effect of LYN knockdown on cell proliferation was analyzed using the WST-1 assay (n = 3; bars, SE; *, *P* < 0.05; Student's t-test). **D.** Cells were treated with dasatinib (DMSO as a control, IC_50_) after LYN knockdown. The effect of LYN depletion on cell migration was determined using a transwell assay.

We next examined whether dasatinib inhibited LYN activity in these sensitive cell lines. We measured phospho-LYN (Y396) levels using an ELISA-based method that specifically detects pY396-LYN. Dasatinib reduced pY396-LYN levels in a concentration-dependent manner in dasatinib-sensitive ADC cell lines (Figure 3A). We also measured pY396-LYN levels in H358 and H1975 cells using an immunoprecipitation assay. To specifically detect pY396-LYN, total LYN proteins were first immunoprecipitated with a LYN antibody and then immunoblotted with an anti-pY416-SRC antibody, which cross-reacts with pY396-LYN. Consistent with the ELISA results, dasatinib reduced pY396-LYN levels in a concentration-dependent manner (Figure 3B). Dasatinib inhibits several kinases, including other SFKs [21, 30]. To determine SFK activation status in LYN-positive lung ADC cells, we used an ELISA-based method to detect tyrosine phosphorylation levels for eight SFKs (SRC, LYN, YES, FYN, LCK, BLK, FGR, and HCK). In H358 and H1975 cells, dasatinib mainly decreased LYN phosphorylation levels in a concentration-dependent manner (Supplementary Figure S3).

SFKs promote cell survival and mitogenesis/transformation by activating the AKT and MAPK pathways, respectively [31, 32]. We therefore examined levels of activated (phosphorylated) AKT and MAPK to evaluate the effects of LYN inhibition on downstream signaling pathways. Dasatinib treatment of H358 cells at concentrations ≥ 1/100 of the IC_50_ value (Supplementary Table S7) decreased both pAKT and pMAPK levels, while higher concentrations of dasatinib increased pMAPK levels. In H1975 cells, dasatinib treatment decreased pMAPK, but not pAKT, levels (Figure 3B).

To assess the effects of LYN depletion on proliferation and migration in dasatinib-sensitive ADC cell lines, H358 and H1975 cells were treated with dasatinib after siRNA-mediated LYN knockdown. LYN knockdown slightly, but significantly, increased proliferation compared to cells transfected with non-targeting (NT) siRNA (H358 and H1975; *P* < 0.05) (Figure 3C). LYN knockdown increased migration in H1975, but not in H358, cells compared to NT siRNA-treated cells (Figure 3D). Together with the finding that dasatinib mainly inhibited active LYN among the SFKs examined (Supplementary Figure S3), these data suggest that LYN might be a major target of dasatinib in lung ADC cells.

**Figure 4:**
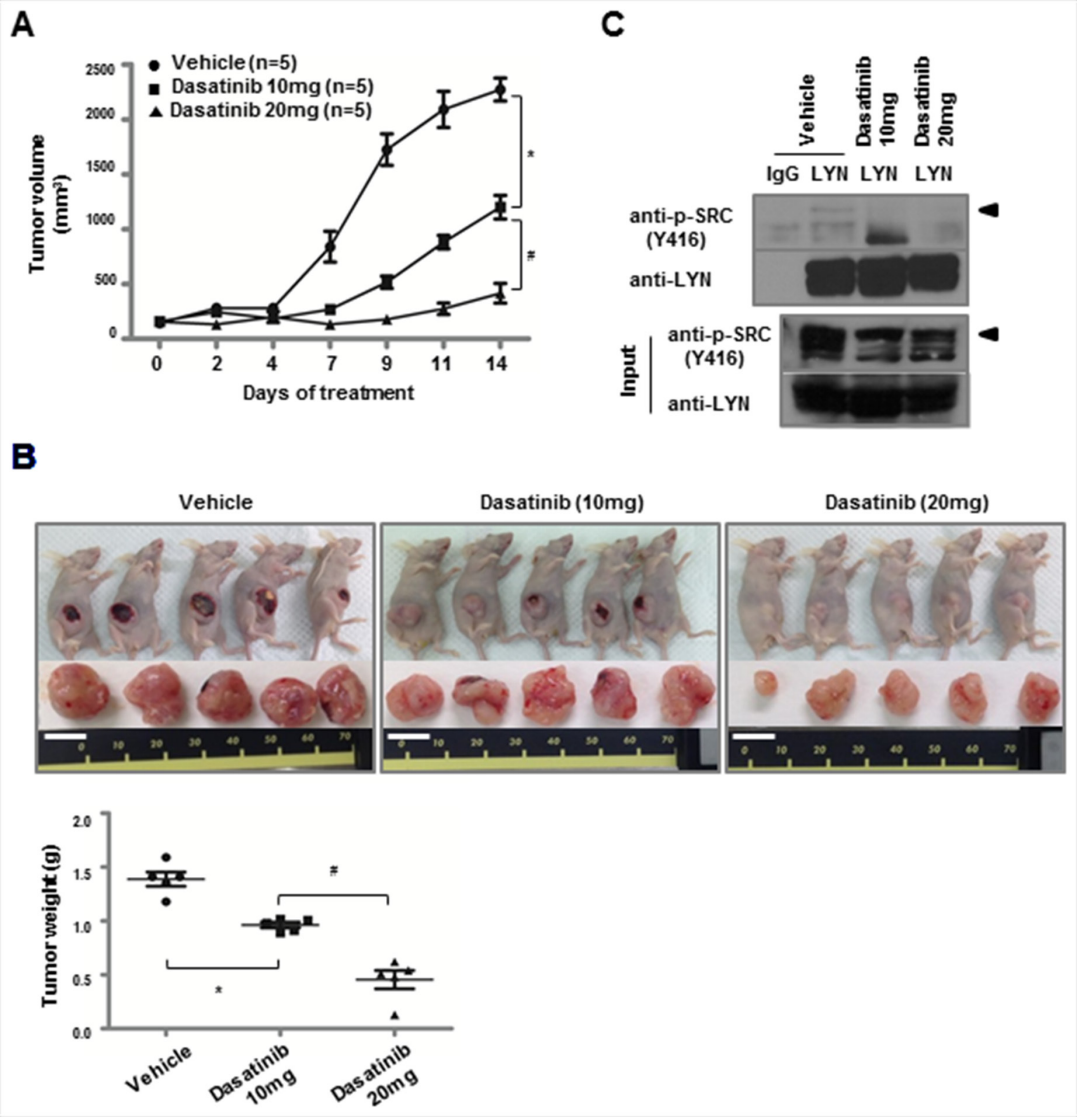
Dasatinib inhibits the growth of LYN-positive lung ADC xenografts **A.** H1975 cells were injected subcutaneously into nude mice. Dasatinib treatment began when the average tumor size reached ~100 mm^3^. Dasatinib was intraperitoneally injected for 2 weeks at either 10 mg·kg^−1^·day^−1^ or 20 mg·kg^−1^·day^−1^ (*, *P* < 0.05 vs. vehicle; ^#^, *P* < 0.05 vs. dasatinib [10 mg]). **B.** H1975 xenografts from (A) were resected, and tumor weights were measured at the time of tumor harvest (bars, mean and SD; *, *P* < 0.05 vs. vehicle; ^#^, *P* < 0.05 vs. dasatinib [10 mg]; white bar = 1 cm). **C.** Immunoprecipitation was performed with an LYN antibody on lysates from H1975 xenograft tumors, followed by immunoblotting with a cross-reactive pY416-Src antibody (◄).

We investigated the effects of dasatinib treatment on lung ADC tumor growth *in vivo* using a nude mouse ADC xenograft model. Tumor growth (i.e., volume and weight) (Figure 4A and 4B) and active pY396-LYN levels were reduced in H1975 xenografts treated with dasatinib, consistent with the results in the H1975 cell line (Figure 4C). However, dasatinib treatment did not inhibit tumor growth in xenografts of H23 cells, which had undetectable LYN expression (Supplementary Figure S4). These data are consistent with the *in vitro* results and confirm that dasatinib inhibits H1975, but not H23, cell viability (Supplementary Figure S1A and S1B).

### SRC and YES are targets for dasatinib in LYN-negative lung ADC cells

**Figure 5:**
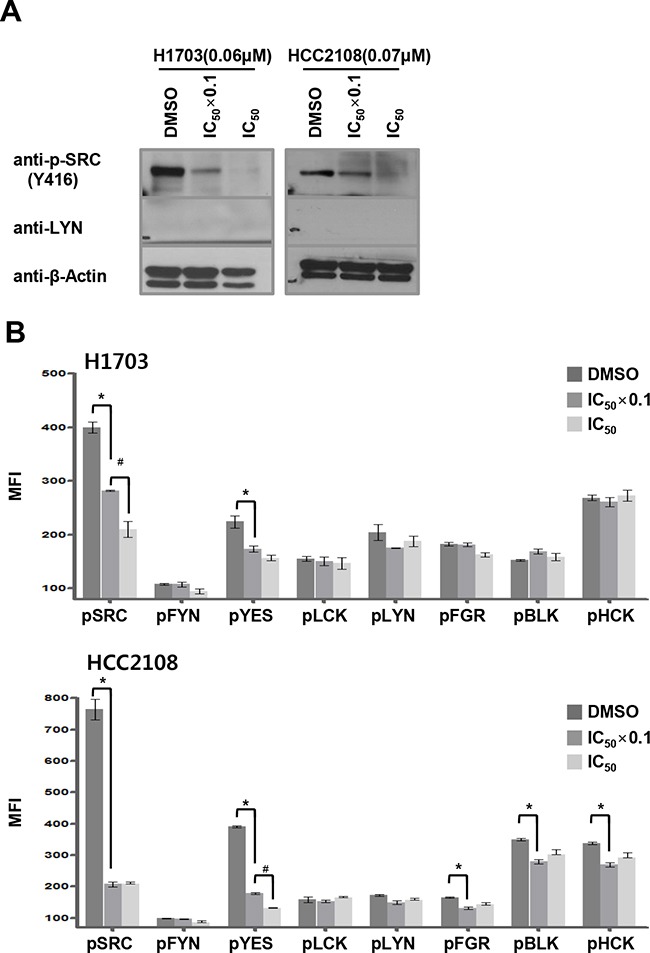
Dasatinib targets SRC and YES in LYN-negative lung ADC cells **A.** Cells were treated with dasatinib at 2 different concentrations (DMSO as control, IC_50_ x 0.1, and IC_50_), and pSFK levels were assessed by immunoblot using a cross-reactive pY416-SRC antibody. **B.** SFK tyrosine phosphorylation levels were quantified using ELISA in dasatinib-treated ADC cells. Cells were treated with dasatinib at 2 different concentrations (DMSO as control, and IC_50_ x 0.1, and IC_50_). The MFI values indicate phospho-Tyr SFK levels (n = 3; bars, SE; *, *P* < 0.05 vs. DMSO; ^#^, *P* < 0.05 vs. IC_50_ × 0.1).

Interestingly, H1703 and HCC2108 cells, in which LYN was undetectable, were highly sensitive to dasatinib treatment, with IC_50_ values of 0.063 and 0.067 μM, respectively (Supplementary Table S5 and Figure 2A). Because dasatinib inhibits several kinases, including other SFKs, we hypothesized that these cells expressed other dasatinib-sensitive SFKs. We detected total levels of activated SFKs using western blots with the cross-reactive anti-pY416-SRC antibody. In both cell lines, dasatinib treatment reduced total pSFK levels in a concentration-dependent manner, suggesting that dasatinib targets other SFKs in these cells (Figure 5A). To identify which SFKs were inhibited, we used the ELISA-based method to detect tyrosine phosphorylation levels for eight SFKs. Dasatinib decreased only SRC and YES phosphorylation levels in a concentration-dependent manner in H1703 and HCC2108 cells, respectively (Figure 5B). These data suggest that other SFK family members, specifically SRC and YES, might be selective targets of dasatinib in LYN-negative ADC cells.

## DISCUSSION

Here, we assessed the clinical significance of LYN overexpression and evaluated the therapeutic potential of treatments targeting LYN in NSCLC. LYN is one of nine SFK family members, which participate in signal transduction and regulate various processes, including growth, survival, migration, and invasion, in cancer cells [4]. The functions of LYN have been investigated primarily in hematopoietic cells, but several recent studies have suggested that LYN plays a critical role in the development and progression of colon, prostate, breast, and lung cancers [15, 16, 18, 20, 33]. Our group previously reported that LYN is among the top epithelial-mesenchymal transition (EMT) signature genes in mesenchymal breast cancer cell lines, and high LYN expression is correlated with lower OS [18]. Su *et al.* found that LYN regulates CD24-induced ERK1/2 activation and that its expression is associated with poor prognosis in colorectal cancer [15]. However, the clinical significance of LYN overexpression has not been characterized in large cohorts of NSCLC patients. In our cohort of 446 NSCLC patients, high LYN expression predicted lower OS in a multivariate analysis. This correlation between high LYN expression and poor OS was stronger for females, non-smokers, and patients with ADC, and was strongest for the non-smoker, female patients with ADC subgroup, in both univariate and multivariate analyses. Although the small sample size should be considered when interpreting these results, our findings suggest that LYN may be a useful prognostic marker, and that pharmacological inhibition of LYN might be an effective clinical strategy for lung cancer patients with ADC, especially in non-smoker female patients.

LYN promotes tumorigenesis; for example, it triggers the initiation of prostate cancer in a regenerated prostate murine model [34]. According to a recent study, EGFR phosphorylates LYN, which then activates MCM7, a critical component for DNA replication, in turn leading to enhanced DNA synthesis and cell growth [35]. In contrast, Sutton *et al.* suggested that LYN functions upstream of EGFR, contributes to constitutive EGFR activation, and consequently increases lung cancer cell viability [20]. In addition, the atypical kinase SgK269 (PEAK1) was identified as a novel LYN kinase substrate that enhances EMT signaling and anchorage-independent growth in basal breast cancer [33]. In this study, LYN knockdown and overexpression experiments revealed that LYN promoted growth/survival, migration, and invasion in lung ADC cells. However, the upstream/downstream regulators of LYN overexpression/activation require further investigation.

Dasatinib is a small-molecule tyrosine kinase inhibitor of both SFKs and ABL and is currently being evaluated in clinical trials for use in many solid tumors, including breast, gastric, pancreatic, head and neck, and lung cancer (www.clinicaltrials.gov). However, recent trials have suggested that dasatinib is not effective in treating NSCLC [9–12, 36]. Haura *et al*. and Johnson *et al*. reported that, despite overall disease control rates of 63% and 43%, only 2 patients (7%) and 1 patient (3%) showed PRs, respectively, in response to dasatinib treatment [9–10]. However, these results are likely due to genetic heterogeneity in the enrolled patient cohorts, and specific NSCLC patient subgroups may benefit from dasatinib therapy. Here, in the overall NSCLC cohort, 50.9% of patients were LYN positive, which was similar to the aforementioned DCR. Most importantly, our study suggests that non-smoker female patients with adenocarcinoma and high LYN expression (27 of 151 ADC cases, 18%) may show PRs to dasatinib therapy, and that this treatment may improve clinical outcomes in these patients.

Based on our finding that LYN expression was associated with clinical outcomes, especially in patients with ADC, we evaluated the efficacy of inhibiting LYN with dasatinib in ADC cell lines and xenografts. Dasatinib treatment inhibited cell growth/viability at IC_50_ values ranging from 0.063 to 0.349 μM in LYN-expressing lung ADC cells. Consistent with these *in vitro* results, dasatinib robustly inhibited growth in H1975 xenograft tumors, which had high LYN levels, but not in H23 xenograft tumors, which did not express LYN. These findings suggest that LYN expression might serve as a theranostic biomarker for dasatinib sensitivity in NSCLC, especially in female non-smoker patients with lung ADC. Johnson *et al*. reported that dasatinib had no effect in patients harboring *EGFR* mutations (deletion of exon 19 and T790M or L858R and T790M) that confer resistance to erlotinib or gefitinib [12]. Recently, Yoshida *etal*. showed that combined treatment with dasatinib and irreversible (afatinib) or T790M-selective (WZ4006) EGFR inhibitors resulted in synergistic antitumor activity in an H1975 cell line harboring T790M and L858R mutations [37]. That report suggests that clinical trials of dasatinib in combination with these EGFR inhibitors are urgently needed. Here, we also found that pAKT and pMAPK levels were reduced when LYN activity was inhibited by dasatinib. In H1975 cells, dasatinib did not decrease AKT phosphorylation (Figure 3B). According to a report by Li *et al*., dasatinib had minimal effects on AKT and MAPK phosphorylation in lung cancer cells expressing *EFGR* double mutations (T790M and L858R) [21]. However, while dasatinib might not affect pAKT levels in H1975 cells, dasatinib treatment combined with AKT inhibition might more potently inhibit cell growth; this possibility requires further investigation. In both H358 and H1975 cells, MAPK phosphorylation increased after dasatinib treatments at concentrations exceeding the IC_50_ value. MAPK activation regulates various cellular mechanisms, such as proliferation, migration, and differentiation [38]. However, many reports paradoxically suggest that activated MAPK can promote cell death in response to various treatments, including anti-cancer drugs [38]. It is possible that the latter effect is due to the restoration of MAPK phosphorylation following high doses of anti-cancer agents.

In a study of lung cancer cell lines using quantitative phosphoproteomics, approximately 40 different kinases were identified as dasatinib targets, including the SFKs LYN, SRC, YES, FYN, and LCK [21]. It is well-established that SRC is necessary for tumor progression in many tumor types, including NSCLC [5]. YES may play a role in retinal microvascular endothelial cells during angiogenesis, and has oncogenic activity in colon carcinoma cells [39–41]. YES and LYN influence the nuclear localization of EGFR via phosphorylation and consequently induce cetuximab resistance in NSCLC cells [42]. Moreover, various SFKs play different roles in the initiation and metastasis of prostate cancer [43]. Our study also suggests that SRC and YES are potential targets of dasatinib treatment in ADCs that lack LYN expression. Additionally, SCR and YES were activated in a mutually exclusive fashion in H1703 and HCC2108 cells, respectively. These findings suggest that the therapeutic potential of treatments targeting SRC and YES in ADC requires further investigation.

## MATERIALS AND METHODS

### Patients and data collection, tissue samples, TMA, and IHC

In total, 446 patients with NSCLC diagnosed at the Samsung Medical Center between 1994 and 2001 were enrolled. The criteria for inclusion in this study included primary NSCLCs that were surgically resected and histopathologically confirmed, no treatment before surgical resection, and the availability of well-preserved paraffin-embedded blocks and clinical information. Baseline characteristics and clinical outcomes from medical records were retrospectively reviewed. Smoking status was defined as either non-smoker (<100 lifetime cigarettes) or smoker. Follow-up periods ranged from 1 to 167 months (mean ± SD, 42.2 ± 32.1 months). Pathologic tumor stages and histological grades of NSCLC were evaluated according to the International Association for the Study of Lung Cancer, American Thoracic Society, and European Respiratory Society guidelines [44]. In tissues with variable histological features, the predominant area was selected to construct the TMA. LYN levels were evaluated in serial sections from TMA blocks by IHC using a mouse monoclonal anti-LYN antibody (Santa Cruz Biotechnology) at a dilution of 1:20. Semi-quantitative assessment of IHC staining was performed by 2 pathologists who were unaware of the clinicopathological details. Membranous staining exceeding 10% was defined as positive.

### Statistical analysis

Correlations between qualitative clinicopathological variables and LYN expression were evaluated using Pearson's c^2^ test or a 2-tailed Fisher's exact test. Overall survival (OS) was measured from the day of NSCLC diagnosis to the day of death due to any cause and was analyzed using the Kaplan–Meier method, with the log-rank test applied for comparisons between groups. The Cox proportional hazards model was used to calculate the relative risks regarding OS rates. The results were adjusted for sex, age, smoking, histology, stage, and LYN expression. SPSS software (SPSS Inc.) was used for statistical analyses. All *p*-values were calculated using Student's *t*-test, and *p*-values less than 0.05 were considered significant.

### Cell culture

Human lung ADC cell lines were obtained from the American Type Culture Collection (H1792) or the Korean Cell Line Bank (H23, H358, H1299, H1703, H1792, H1975, HCC1171, HCC2108, and SK-LU-1). All cells were grown at 37°C with 5% CO_2_ in RPMI-1640 or Dulbecco's modified Eagle's medium (HyClone) containing 10% fetal bovine serum (HyClone) and 1% penicillin/streptomycin (HyClone). All cells were monitored for *Mycoplasma* contamination using a PCR-based method for detection. Detected *Mycoplasma* were eliminated using BM cyclins (Roche).

### Western blot analysis

Cells were harvested and lysed with RIPA buffer containing a phosphatase inhibitor cocktail and a protease inhibitor cocktail tablet (Roche). Primary anti-LYN and anti-β-actin (Santa Cruz Biotechnology), anti-FLAG (Sigma), anti-SRC, anti-pSRC, anti-AKT, anti-pAKT, anti-MAPK, and anti-pMAPK (Cell Signaling) antibodies were used at a 1:1000 dilutions. Proteins were detected using ECL solution (Millipore).

### siRNA silencing, gene overexpression, and cell proliferation, migration, and invasion assays

siRNAs against LYN and a non-targeting SMART pool were purchased from Thermo Scientific. FLAG-LYN and FLAG overexpression tags were constructed using standard molecular cloning methods. Briefly, 2 × 10^6^ cells were transfected with siRNAs (20 nmol) or overexpression constructs (10 μg) for 72 h using siLentFect reagent (Bio-Rad) or Lipofectamine (Invitrogen), respectively. The effect of gene silencing or overexpression on cell proliferation was measured using the WST-1 cell viability assay kit (Daeil Lab Service). Motility and invasion were quantified by QCMTM 24-well cell migration or invasion assays according to the manufacturer's instructions (Millipore). All assays were performed in triplicate, and the means and standard errors are shown.

### Drug treatment and cell viability assay

Dasatinib (Selleckchem) was obtained in powder form, reconstituted in dimethyl sulfoxide (DMSO) at 100 mM, and used at indicated concentrations. Cell viability was monitored using the WST-1 assay kit. All experiments were performed in triplicate, and IC_50_ values were calculated using Prism 5.0 software (GraphPad).

### ELISA

Cell lines were treated with dasatinib, and total protein was extracted using the Milliplex Map 8-Plex Human SRC Family Kinase Phosphoprotein, Milliplex Map Phospho-LYN (Tyr397), and MAPmate kits (Millipore) according to the manufacturer's instructions. Median fluorescence intensities were measured using a Bio-Plex 200 system (Bio-Rad). All assays were performed in triplicate, and the means and standard errors are shown.

### Tumor xenograft treatment

Female nude mice were injected subcutaneously with H23 (2 × 10^6^) or H1975 (3 × 10^6^) cells. After 4 weeks, mice bearing tumors of ~200 mm^3^ (H23) and ~100 mm^3^ (H1975) were randomly treated with vehicle (5% Chremophor EL in physiological saline solution) or dasatinib (10 mg·kg^−1^·day^−1^ or 20 mg·kg^−1^·day^−1^) intraperitoneally for 2 weeks. Tumor diameters were measured using a digital caliper two to three times per week, and tumor sizes were calculated using the following formula: (3.14/6) (length × width^2^).

## SUPPLEMENTARY FIGURES AND TABLES




