# Supplementary material for: LYN expression predicts the response to dasatinib in a subpopulation of lung adenocarcinoma patients
Source: Oncotarget. 2016 Oct 14;7(50):82876–88. doi: 10.18632/oncotarget.12657 (PMC5347739; doi:10.18632/oncotarget.12657)
Supplement: Supplementary file 1 [file oncotarget-07-82876-s001.pdf]

LYN expression predicts the response to dasatinib in a subpopulation of lung adenocarcinoma patients

SUPPLEMENTARY FIGURES AND TABLES

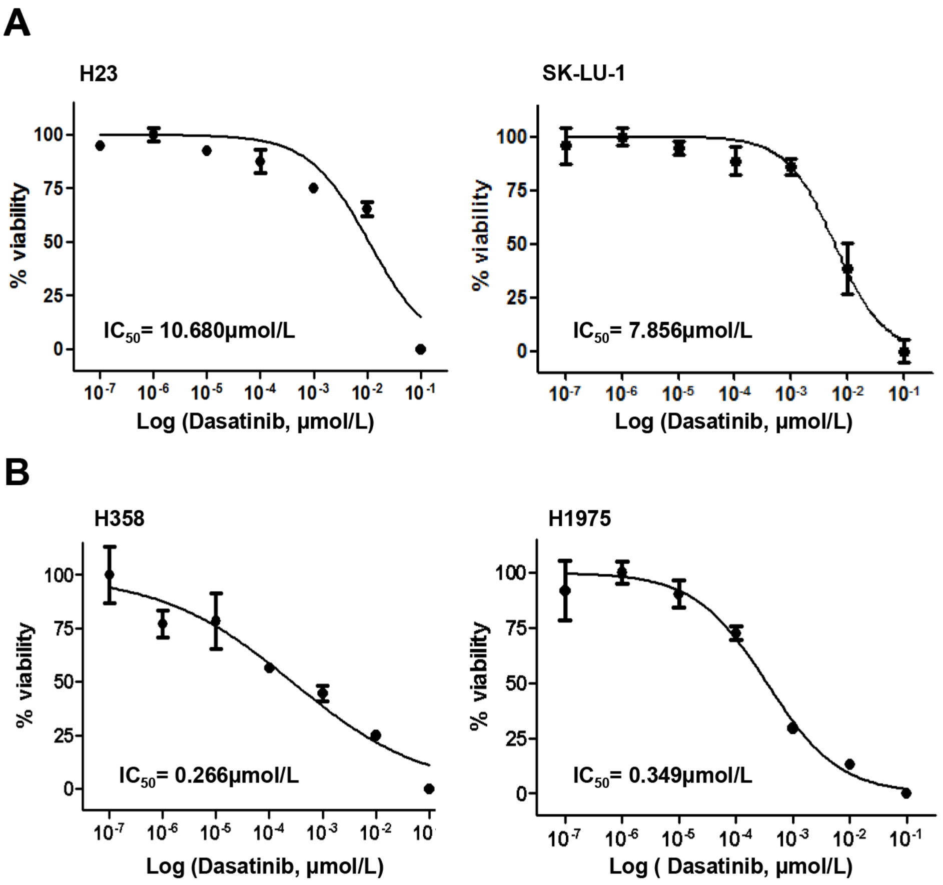

Supplementary Figure S1: Dasatinib inhibits viability of lung ADC cells expressing high levels of LYN. Inhibitory effects of dasatinib on cell viability were assessed by dose-response curves in **A.** low LYN-expressing cells (H23 and SK-LU-1) and **B.** high LYN-expressing cells (H358 and H1975). IC50 values were calculated based on fits using sigmoidal curves (n = 3).

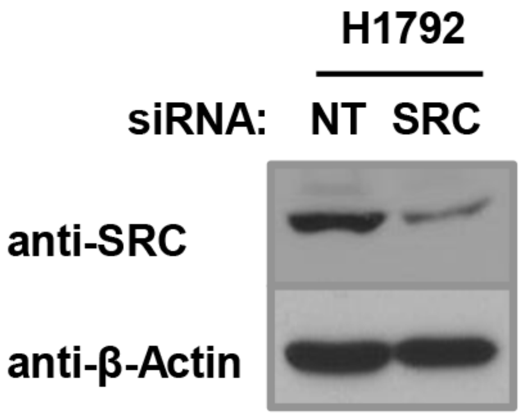

Supplementary Figure S2: Specificity of the SRC antibody (32G6 clone) is confirmed by western blot analysis using siRNA. Knockdown of SRC by siRNAs was confirmed by western blot analysis in H1792 cells. NT, non-targeting.

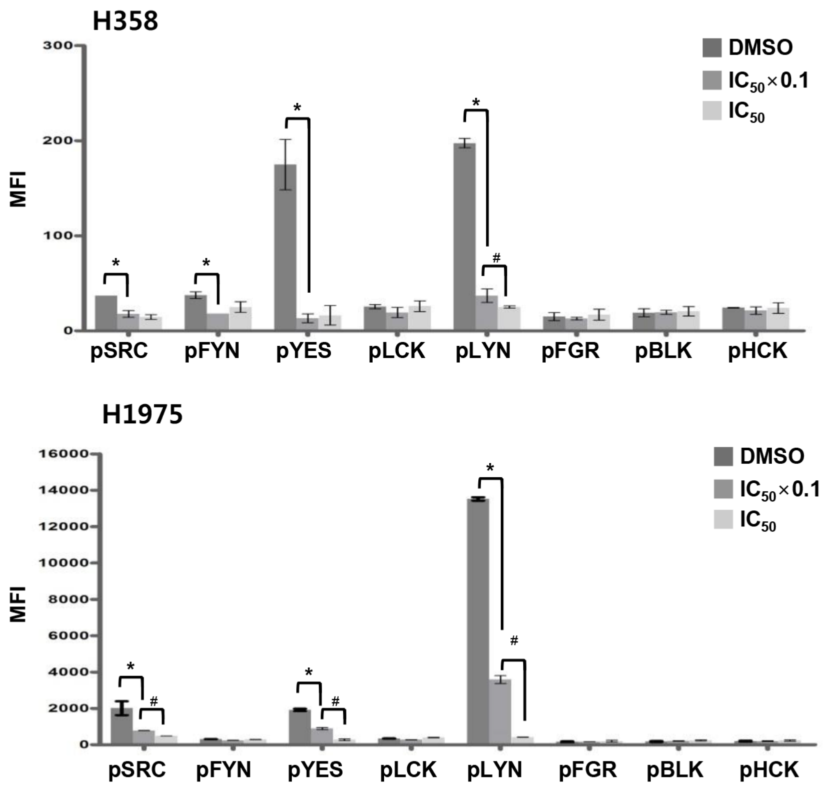

Supplementary Figure S3: LYN might be a major target for dasatinib in LYN-positive lung ADC cells. Tyrosine phosphorylation levels of activated SFKs were quantified in dasatinib-treated H358 and H1975 cells using ELISA. Cells were treated with dasatinib at 2 varying concentrations (DMSO as control, and $IC_{50} \times 0.1$, and IC_{50}). The MFI values indicate the level of phospho-Tyr of SFKs (n = 3; Bars, SE; *, $P < 0.05$ vs. DMSO; #, $P < 0.05$ vs. $IC_{50} \times 0.1$).

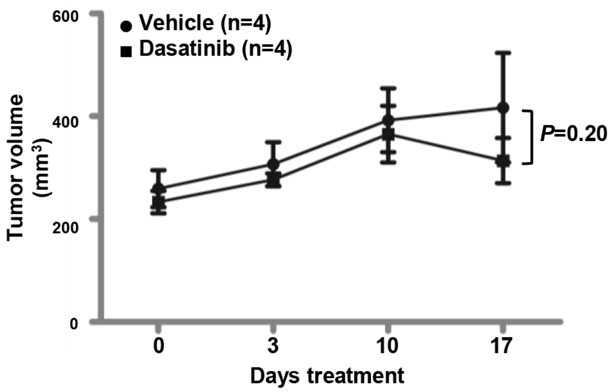

Supplementary Figure S4: Dasatinib did not inhibit the growth of LYN-negative lung ADC xenografts. H23 cells were injected subcutaneously into nude mice. Dasatinib treatment for H23 xenografts were initiated when the average tumor size reached ~200 mm^3. Dasatinib was intraperitoneally injected for 2 weeks at a dosage of 10 $mg \cdot kg^{-1} \cdot day^{-1}$.

Supplementary Table S1: Clinicopathologic features of NSCLC patients according to the LYN expression status

Variable Category	No (%)	LYN (+) (%) n=227(50.9)	LYN (-) (%) n=219(49.1)	*P*
Sex				0.293
Male	360(80.7)	186(81.9)	174(79.5)	
Female	86(19.3)	41(18.1)	45(20.5)	
Age				0.313
≥65	165(37.0)	81(35.7)	84(38.4)	
<65	281(63.0)	146(64.3)	135(61.6)	
Smoking				0.410
Smoker	327(73.3)	168(74.0)	159(72.6)	
Non-smoker	119(26.7)	59(26.0)	60(27.4)	
Histology				0.705
SCC	254(57.0)	126(55.5)	128(58.4)	
ADC	151(33.8)	82(35.7)	70(32.0)	
Others	41(9.2)	20(8.8)	21(9.6)	
Lymph node				0.165
Negative	286(64.1)	151(66.5)	135(61.6)	
Positive	160(35.9)	76(33.5)	84(38.4)	
Stage				0.605
I	246(55.2)	130(57.3)	116(53.0)	
II	102(22.8)	48(21.1)	54(24.7)	
III	98(22.0)	49(21.6)	49(22.4)	

SCC, squamous cell carcinoma; ADC, adenocarcinoma; *P*, *p*-value

Supplementary Table S2: Univariate analysis of prognostic factors for overall survival

Variable	Category	Univariate		
		HR	95% CI	P
Sex	Male/Female	2.055	1.360 - 3.105	0.001
Age group	≥65 yr/<65 yr	1.505	1.137 - 1.992	0.004
Smoking	Smoker /Non-smoker	1.458	1.047 - 2.032	0.026
Histology	SCC/ADC	1.001	0.723 - 1.215	0.328
Stage	III/I+II	2.013	1.714 - 2.365	<0.01
LYN	Positive/Negative	1.186	0.899 - 1.564	0.228

HR, hazard ratio; CI, confidence interval

Supplementary Table S3: Multivariate analysis of prognostic factors for overall survival by NSCLC subgroups

Variable	Category	Female (n=86)			Non-smoker (n=119)		
		HR	95% CI	*P*	HR	95% CI	*P*
Sex	Male/Female	-	-	-	1.650	0.754 - 3.614	0.210
Age group	≥65 yr/<65 yr	1.471	0.577 -3.748	0.419	2.087	1.045 - 4.166	0.037
Smoking	Smoker /Non-smoker	0.406	0.073 - 2.244	0.406	-	-	-
Stage	III/I+II	7.855	2.256 - 9.354	0.001	3.083	1.326 - 7.171	0.009
LYN	Positive/Negative	2.097	0.919 - 4.790	0.079	2.527	1.323 - 4.828	0.005

Variable	Category	ADC (n=151)		
		HR	95% CI	*P*
Sex	Male/Female	1.825	0.859 - 3.880	0.118
Age group	≥65 yr/<65 yr	1.372	0.827 - 2.278	0.221
Smoking	Smoker /Non-smoker	1.579	0.814 - 3.063	0.177
Stage	III/I+II	5.105	2.860 - 9.113	<0.01
LYN	Positive/Negative	1.309	0.808 - 2.122	0.274

Supplementary Table S4: *LYN* and *EGFR* mutation data in the intersection subgroup (FNA, in Figure 1C).

See Supplementary File 1

Supplementary Table S5: The raw data and *p*-value corresponding to Figure 2B

Application	H358			H1792			H1975		
	NT	LYN	*p*-value	NT	LYN	*p*-value	NT	LYN	*p*-value
Proliferation (OD_{450})	0.6900	0.7250		0.8904	0.7030		0.6658	0.5633	
	0.6900	0.7750	0.069	1.0438	0.7073	0.002	0.7520	0.5527	0.002
				0.9794	0.6980		0.7118	0.5694	
Migration (OD_{570})	0.43977	0.2807		0.0680	0.0618		0.0617	0.0600	
	0.40501	0.3568	0.007	0.0688	0.0613	0.001	0.0625	0.0606	0.002
	0.41929	0.3166		0.0682	0.0637		0.0623	0.0604	
Invasion (OD_{570})	0.30743	0.2822		0.0635	0.0063		0.0770	0.0702	
	0.27559	0.2641	0.062	0.0646	0.0635	0.101	0.0767	0.0706	0.001
	0.29095	0.2484		0.0647	0.0636		0.0769	0.0709	

Supplementary Table S6: EGFR mutation status of lung ADC cells from Cancer Cell Line Encyclopedia (CCLE) data analysis

Cell line	EGFR mutation
H23	WT
SK-LU-1	WT
H358	WT
H1299	WT
H1703	WT
H1792	WT
H1975	L858R+T790M
HCC1171	WT
HCC2108	WT

Supplementary Table S7: IC50 values of dasatinib in lung ADC cells

Cell line	Dasatinib IC_{50} (μmol/L)	Response
H23	10.68	Resistant (>0.6 μmol/L)
SK-LU-1	7.856	Resistant
H358	0.266	Sensitive
H1299	0.236	Sensitive
H1703	0.063	Sensitive
H1792	0.125	Sensitive
H1975	0.349	Sensitive
HCC1171	0.308	Sensitive
HCC2108	0.067	Sensitive
