# Supplementary material for: LYN expression predicts the response to dasatinib in a subpopulation of lung adenocarcinoma patients
Source: Oncotarget. 2016 Oct 14;7(50):82876–88. doi: 10.18632/oncotarget.12657 (PMC5347739; doi:10.18632/oncotarget.12657)
Supplement: Supplementary file 2 [file oncotarget-07-82876-s002.docx]

**Supplementary Table 4. *LYN* and *EGFR* mutation data in the intersection subgroup (FNA, in Figure 1C)**

| **Sample name** | **LYN expression**  **(+/–)** | ***LYN* exon 8** | | ***LYN* exon 11** | ***LYN* exon 12** | ***EGFR*  exon 19** | ***EGFR* exon 20** | ***EGFR* exon 21** | |
| --- | --- | --- | --- | --- | --- | --- | --- | --- | --- |
|  |  | **E241D** | **G264S** | **Y357C** | **K404N** | **ΔE746-A750** | **T790M** | | **L858R** |
| FNA01 | **+**  **(N=27)** | WT | WT | WT | WT | WT | WT | | WT |
| FNA02 |  | WT | WT | WT | WT | WT | WT | | WT |
| FNA03 |  | WT | WT | WT | WT | WT | WT | | L858R |
| FNA04 |  | WT | WT | WT | WT | WT | WT | | WT |
| FNA05 |  | N/A | N/A | N/A | N/A | WT | N/A | | N/A |
| FNA06 |  | WT | WT | WT | WT | WT | WT | | WT |
| FNA07 |  | WT | WT | N/A | WT | WT | WT | | L858R |
| FNA08 |  | WT | WT | N/A | WT | WT | WT | | L858R |
| FNA09 |  | WT | WT | WT | WT | WT | WT | | WT |
| FNA10 |  | WT | WT | WT | WT | WT | WT | | WT |
| FNA11 |  | WT | WT | Y357C | WT | WT | WT | | WT |
| FNA12 |  | WT | WT | WT | WT | WT | WT | | WT |
| FNA13 |  | WT | WT | N/A | WT | WT | WT | | L858R |
| FNA14 |  | WT | WT | WT | WT | WT | WT | | WT |
| FNA15 |  | WT | WT | WT | WT | WT | WT | | WT |
| FNA16 |  | WT | WT | WT | WT | ΔE746-A750 | WT | | WT |
| FNA17 |  | WT | WT | WT | WT | ΔE746-A750 | WT | | WT |
| FNA18 |  | WT | WT | WT | WT | WT | WT | | L858R |
| FNA19 |  | WT | WT | WT | WT | WT | WT | | WT |
| FNA20 |  | WT | WT | WT | WT | WT | WT | | L858R |
| FNA21 |  | N/A | | | | | | | |
| FNA22 |  |  |  |  |  |  |  |  |  |
| FNA23 |  |  |  |  |  |  |  |  |  |
| FNA24 |  |  |  |  |  |  |  |  |  |
| FNA25 |  |  |  |  |  |  |  |  |  |
| FNA26 |  |  |  |  |  |  |  |  |  |
| FNA27 |  |  |  |  |  |  |  |  |  |

| FNA28 | **–**  **(N=41)** | WT | WT | WT | WT | WT | WT | WT |
| --- | --- | --- | --- | --- | --- | --- | --- | --- |
| FNA29 |  | WT | WT | WT | WT | WT | WT | WT |
| FNA30 |  | WT | WT | WT | WT | WT | WT | WT |
| FNA31 |  | WT | WT | WT | WT | WT | WT | L858R |
| FNA32 |  | WT | WT | WT | WT | WT | WT | L858R |
| FNA33 |  | WT | WT | N/A | WT | WT | WT | L858R |
| FNA34 |  | WT | WT | N/A | N/A | WT | WT | WT |
| FNA35 |  | WT | WT | WT | WT | WT | WT | L858R |
| FNA36 |  | WT | WT | N/A | WT | WT | WT | WT |
| FNA37 |  | WT | WT | WT | WT | WT | WT | WT |
| FNA38 |  | WT | WT | WT | WT | WT | WT | L858R |
| FNA39 |  | WT | WT | WT | WT | WT | WT | L858R |
| FNA40 |  | WT | WT | WT | WT | WT | WT | L858R |
| FNA41 |  | WT | WT | WT | WT | WT | WT | WT |
| FNA42 |  | WT | WT | WT | WT | WT | WT | L858R |
| FNA43 |  | WT | WT | WT | WT | WT | WT | WT |
| FNA44 |  | WT | WT | WT | WT | WT | WT | WT |
| FNA45 |  | WT | WT | WT | WT | WT | WT | WT |
| FNA46 |  | WT | N/A | N/A | WT | WT | WT | WT |
| FNA47 |  | WT | WT | WT | WT | WT | WT | WT |
| FNA48 |  | WT | N/A | WT | WT | N/A | N/A | L858R |
| FNA49 |  | WT | WT | WT | WT | WT | N/A | L858R |
| FNA50 |  | WT | WT | WT | WT | WT | WT | WT |
| FNA51 |  | WT | WT | WT | WT | WT | WT | WT |
| FNA52 |  | WT | WT | WT | WT | WT | WT | WT |
| FNA53 |  | WT | WT | N/A | WT | WT | WT | L858R |
| FNA54 |  | WT | WT | WT | WT | WT | WT | WT |
| FNA55 |  | WT | N/A | WT | WT | WT | N/A | L858R |
| FNA56 |  | WT | WT | N/A | WT | WT | WT | L858R |
| FNA57 |  | WT | WT | WT | WT | ΔE746-A750 | WT | WT |
| FNA58 |  | WT | WT | WT | WT | WT | WT | WT |
| FNA59 |  | WT | WT | WT | WT | WT | WT | WT |
| FNA60 |  | WT | WT | WT | WT | WT | WT | WT |
| FNA61 |  | WT | WT | WT | WT | WT | WT | WT |
| FNA62 |  | N/A | | | | | | |
| FNA63 |  |  |  |  |  |  |  |  |
| FNA64 |  |  |  |  |  |  |  |  |
| FNA65 |  |  |  |  |  |  |  |  |
| FNA66 |  |  |  |  |  |  |  |  |
| FNA67 |  |  |  |  |  |  |  |  |
| FNA68 |  |  |  |  |  |  |  |  |

EGFR, epidermal growth factor receptor; WT, wild type; N/A, not applicable

| **Chi-square test** | ***LYN* exon 8** | | ***LYN* exon 11** | ***LYN* exon 12** | ***EGFR*  exon 19** | ***EGFR* exon 20** | ***EGFR* exon 21** | |
| --- | --- | --- | --- | --- | --- | --- | --- | --- |
| **LYN+/–** | **E241D** | **G264S** | **Y357C** | **K404N** | **ΔE746-A750** | **T790M** | | **L858R** |
| *p*-value | N/A | N/A | 0.181 | N/A | 0.274 | N/A | | 0.628 |
